# 1213. The Effect of a Prospective Intervention Program with Automated Monitoring on Hand Hygiene Performance in Long-term and Acute Care Units at a Veteran Affairs Medical Center

**DOI:** 10.1093/ofid/ofac492.1045

**Published:** 2022-12-15

**Authors:** James W Arbogast, Pamela Wagner, Susan E Mahrer, Vanessa Christian, Barbara L Lane, V Lorraine Cheek, Gregory A Robbins, W Grant Starrett, Albert E Parker, John M Boyce, Hari Polenakovik

**Affiliations:** GOJO Industries, Inc., Akron, Ohio; GOJO Industries, Inc., Akron, Ohio; Dayton VA Medical Center, Dayton, Ohio; Dayton VA Medical Center, Dayton, Ohio; Dayton VA Medical Center, Dayton, Ohio; Dayton VA Medical Center, Dayton, Ohio; GOJO Industries, Inc., Akron, Ohio; Dayton VA Medical Center, Dayton, Ohio; Montana State University, Bozeman, Montana; JM Boyce Consulting, LLC, Middletown, Connecticut; Dayton VA Medical Center, Dayton, Ohio

## Abstract

**Background:**

There is emerging evidence that implementation of an automated hand hygiene monitoring system (AHHMS) must be part of a multimodal hand hygiene (HH) program that includes complementary strategies. There are few published studies describing in detail the intervention strategies used with an AHHMS.

**Methods:**

An AHHMS that provides group HH performance rates (100 x HH product dispenses divided by the number of room entries plus exits) was implemented on two Acute Care (AC) units and six long-term care (LTC) units at a Veterans Affairs Medical Center from March 2021 through April 2022. After a 4-week baseline period and 2.5-week washout period, the 52-week intervention period included many components, such as weekly huddles, unit nurse manager engagement, vendor provided clinician-based training and feedback, leadership support, unit recognition, signage and development of a new slogan to remind colleagues to perform HH. Statistical analysis was performed with a Poisson general additive mixed model.

**Results:**

During the 4-week baseline period, the median HH performance rate was 18.6 (95% CI: [16.5, 21.0]) for all 8 units. During the intervention period, the median HH rate increased to 21.6 [19.1, 24.4], and during the last 4 weeks of the intervention period (exactly 1 year after baseline), the 8 units exhibited a median HH rate of 25.1 [22.2, 28.4], (*p* < 0.0001) [Figure 1]. The median HH rate increased from 17.5 to 20.0 (*p* < 0.0001) in LTC units and from 22.9 to 27.2 (p < 0.0001) in AC units. The intervention increased the use of hand sanitizer from 57.5% during baseline to 65.1% (*p* < 0.0001). The increase in HH rates was due to HH events increasing from 88,758 dispenses during the baseline to 123,722 dispenses during the last 4 weeks of the intervention. Direct observation results during the same periods showed HH compliance ranging from 61-86%.

**Figure 1:**
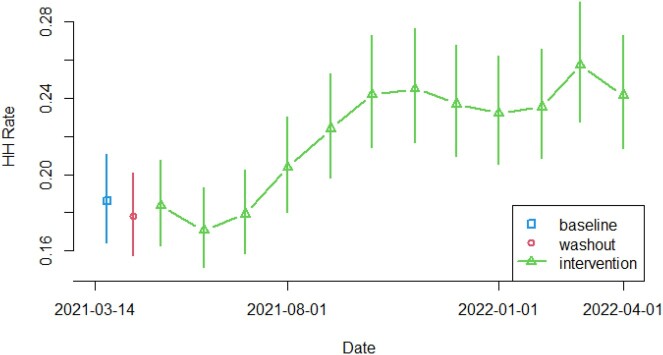
- Monthly Hand Hygiene Performance Rates for all Units

The green curve shows the change in the median HH rate during the intervention period compared to the baseline and washout periods, with vertical bars showing 95% confidence intervals for the monthly HH rate.

**Conclusion:**

The intervention increased hand sanitizer usage and HH performance rates for all units. AC units were consistently better than LTC units, which have more visitors and more mobile veterans. Further HH improvement will rely on continued implementation of complementary strategies and long-term monitoring.

**Disclosures:**

**James W. Arbogast, PhD**, GOJO Industries, Inc.: employee **Pamela Wagner, RN MSN CPPS**, GOJO Industries, Inc: Employee of GOJO **Gregory A. Robbins, BS**, GOJO Industries, Inc.: Current employee of GOJO Industries, Inc. **Albert E. Parker, PhD**, GOJO Industries: Advisor/Consultant **John M. Boyce, MD**, Diversey: Advisor/Consultant|Diversey: Expert Testimony|Diversey: Travel support|GOJO Industries: Advisor/Consultant|GOJO Industries: Expert Testimony|GOJO Industries: Travel support|Sodexo Healthcare: Advisor/Consultant.

